# Supplementary material for: Trans-lesion synthesis and mismatch repair pathway crosstalk defines chemoresistance and hypermutation mechanisms in glioblastoma
Source: Nat Commun. 2024 Mar 4;15:1957. doi: 10.1038/s41467-024-45979-5 (PMC10912752; doi:10.1038/s41467-024-45979-5)
Supplement: Supplementary file 1 — Supplementary Information [file 41467_2024_45979_MOESM1_ESM.pdf]

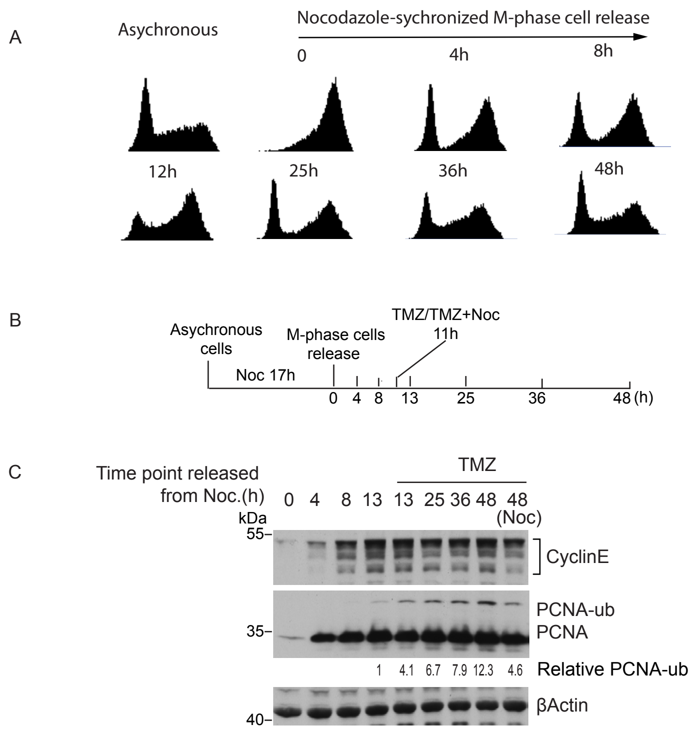

Supplementary Figure 1. **TMZ induced PCNA mono-ubiquitination in nocodazole-synchronized U373 cells**

(A) Cell cycle profiles of asynchronous cells, nocodazole-arrested cells, and cells at different times following release from metaphase arrest.
(B) Scheme illustrating design of experiment to test effect of TMZ-treatment on PCNA mono-ubiquitination in synchronized cells.
(C) Immunoblot showing levels of mono-ubiquitinated PCNA and cyclin E (a late G1/S-phase cell cycle marker) in nocodazole-synchronized cells. 50 μMTMZ was added conditionally to some cultures 11 h following release from the nocodazole block.

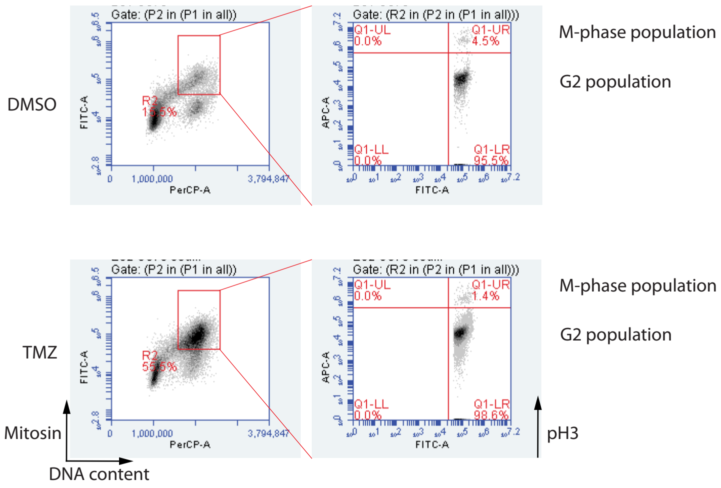

Supplementary Figure 2. Gating strategy for detecting and quantifying mitosin + phospho-histone H3 doubly-stained cells.

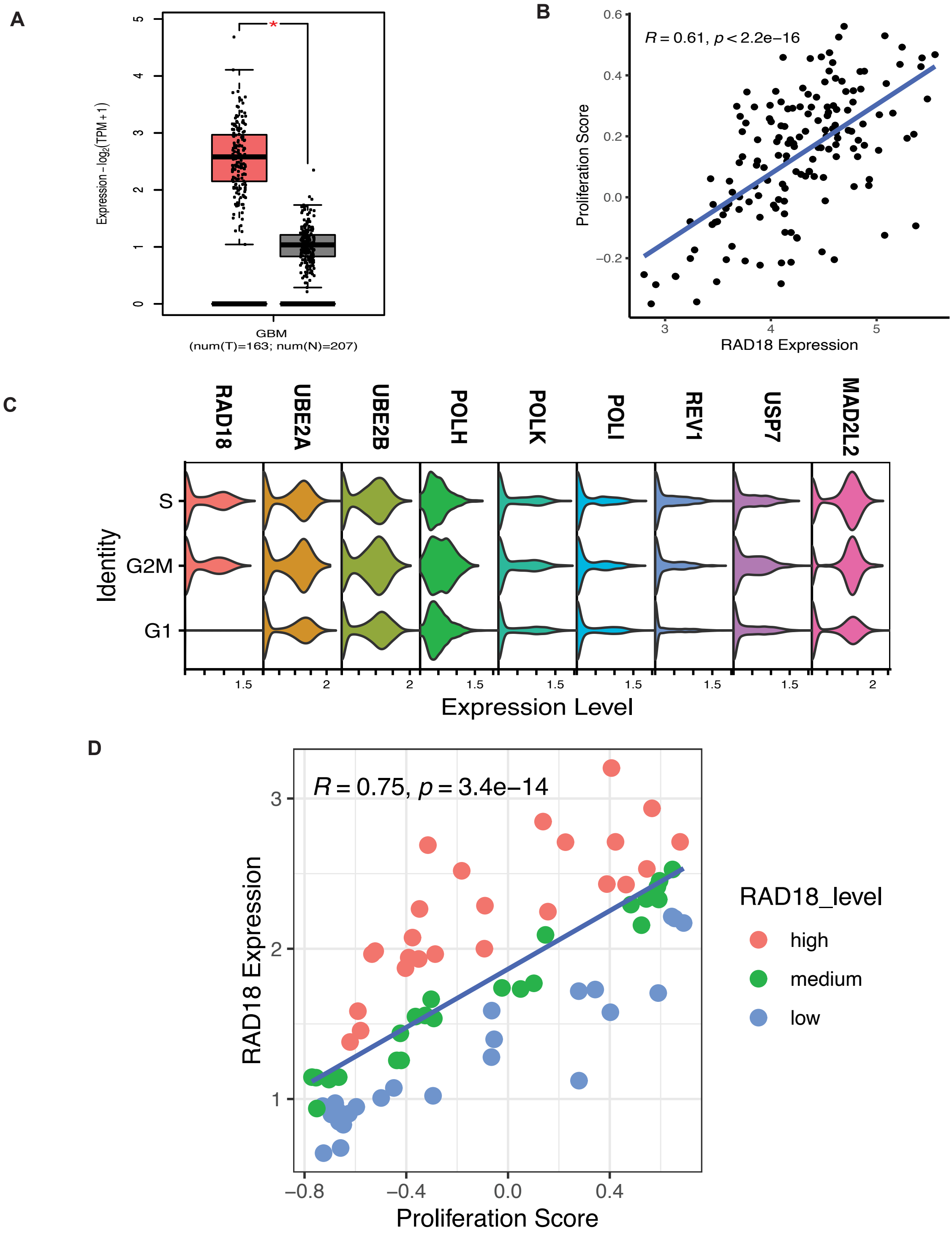

Supplementary Figure 3. Analysis of RAD18 expression in patient-derived glioblastomas

(A) Comparison of RAD18 mRNA expression levels in GBM vs. normal tissue of TCGA data (from GEPIA2 [Supplementary References 1]). Box plots show the lower and upper quartiles. Red: GBM; Gray: normal tissue.
(B) Linear regression showing positive correlation between RAD18 expression and proliferation score in GBM (using TCGA data). The Pearson correlation coefficient is shown. Source data are provided.
(C) Violin plots showing expression levels of RAd18 and other TLS pathway-related genes in malignant glioma. Source data are provided.
(D) Dot plot showing correlation between RAD18 expression and proliferation score in recurrent GBM tumors. Samples were stratified according to RAD18 expression by tertiles. Spearman's rank correlation coefficient is indicated.

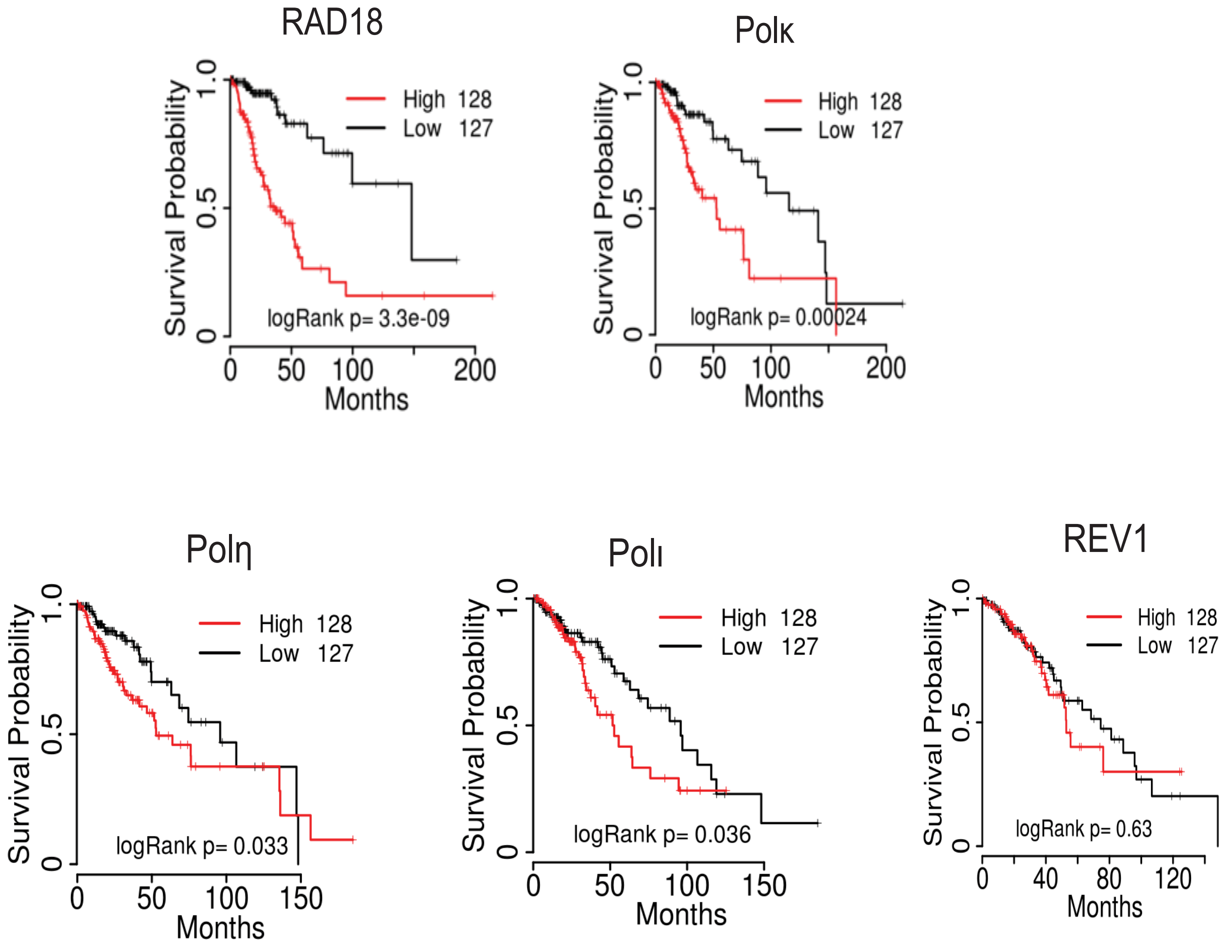

Supplementary Figure 4. Correlation between expression levels of TLS pathway genes and glioma patient survival. Kaplan-Meier curves showing survival of LGG patients from TCGA stratified by high expression (upper quartile, n=128) and low expression (bottom quartile, n=127) of TLS pathway genes. P values were determined by Log-rank test.

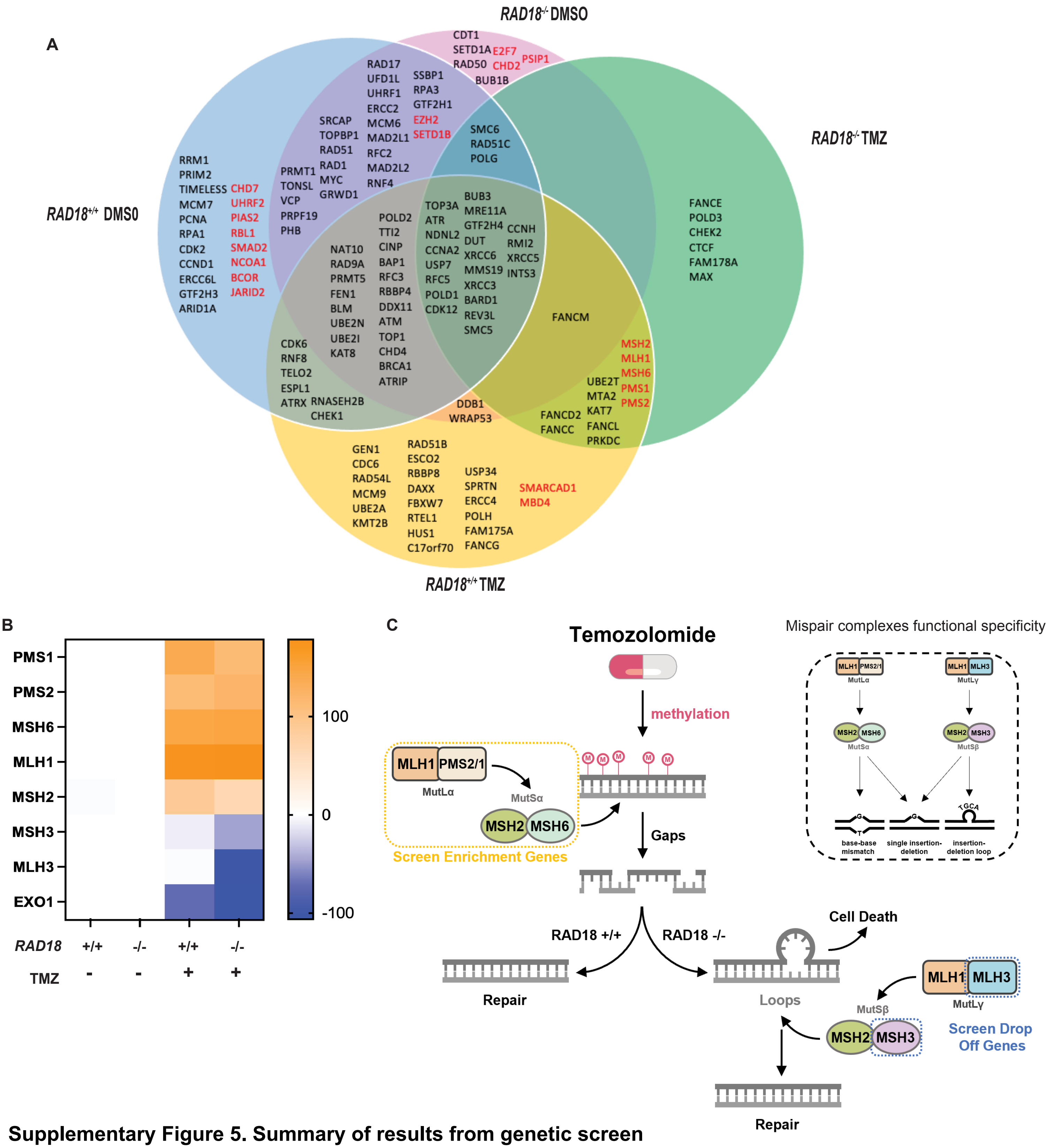

Supplementary Figure 5. Summary of results from genetic screen

(A) Venn diagram showing sgRNAs targeting DDR genes that were significantly dropped-out (black) or enriched (red) in different genotypes (RAD18+/+, RAD18-/-), grown in the presence of TMZ or DMSO (for no-drug control group)
(B) Heatmap showing relative enrichment or dropout of sgRNAs targeting MMR genes in different experimental groups.
(C) Proposed mechanism for differential dropout of MMR pathway genes in TMZ-treated RAD18-/- cells. According to this scheme, RAD18-deficiency induces DNA loops that are repaired in a manner involving the MMR loop-resolving complex factors MLH1, MSH3, but not other MMR proteins

Supplementary Figure 6

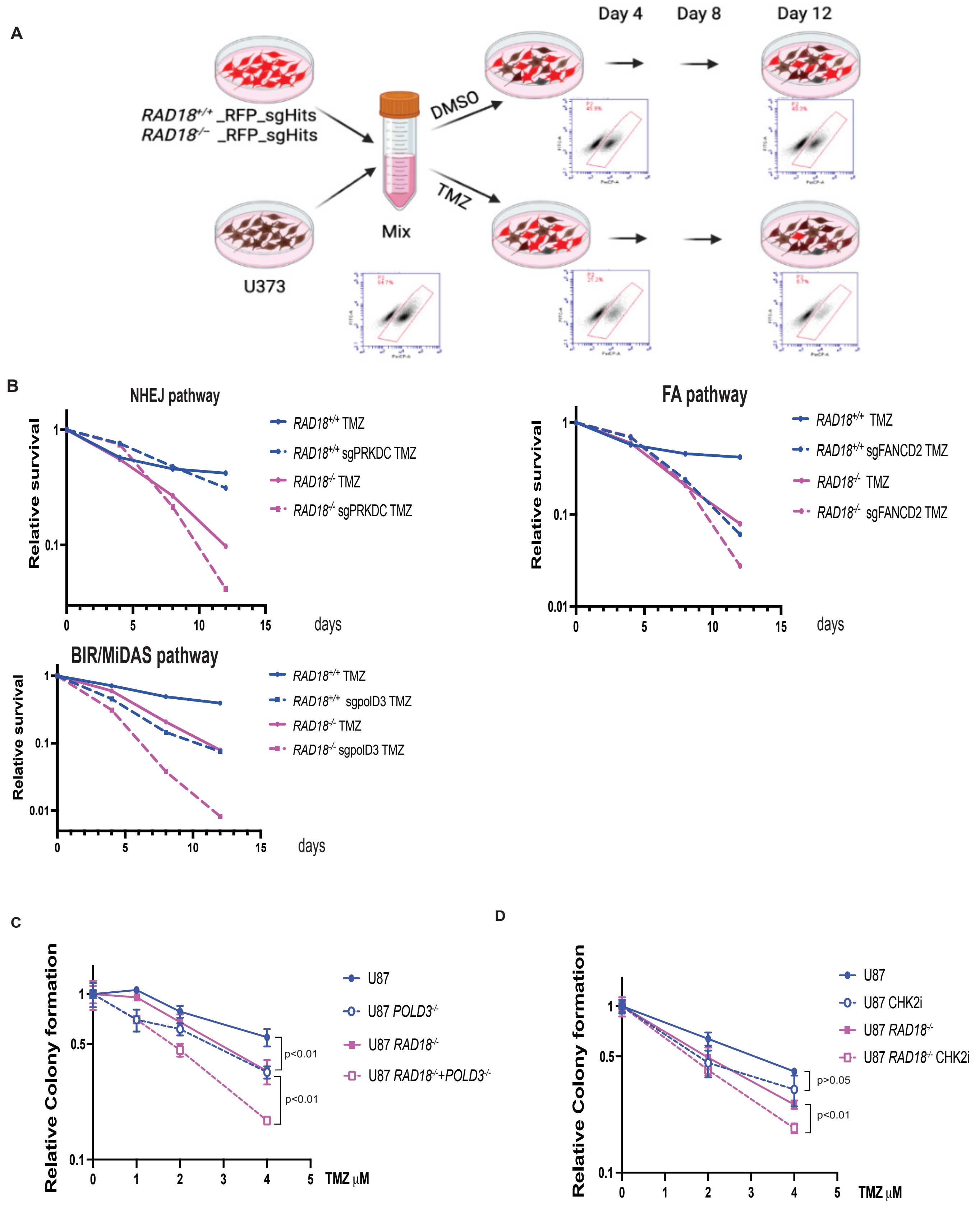

Supplementary Figure 6. Validation of genetic screen for determinants of TMZ-sensitivity in GBM

(A) Experimental workflow of fluorescence-based competitive growth assays
(B) Results of competitive growth assays showing TMZ-sensitivities of *FANCD2*, *POLD3* and *PRKDC*- deficient cells.
(C) Clonogenic survival assays showing TMZ-sensitivity of WT, *RAD18*$^{-/-}$, *POLD3*$^{-/-}$ and *RAD18*$^{-/-}$ *POLD3*$^{-/-}$ U87 cells.
(D) Clonogenic survival assays showing TMZ-sensitivity of WT and *RAD18*$^{-/-}$ U87 cells when grown in the presence or absence of CHK2 inhibitor (ChK2i). TMZ and CHK2i were replenished daily for 5 days.
All data points of (C) and (D) represent mean of 3 replicates ± SD; p values were determined by two-sided t test. (C) was created with BioRender.com

Supplementary Figure 7

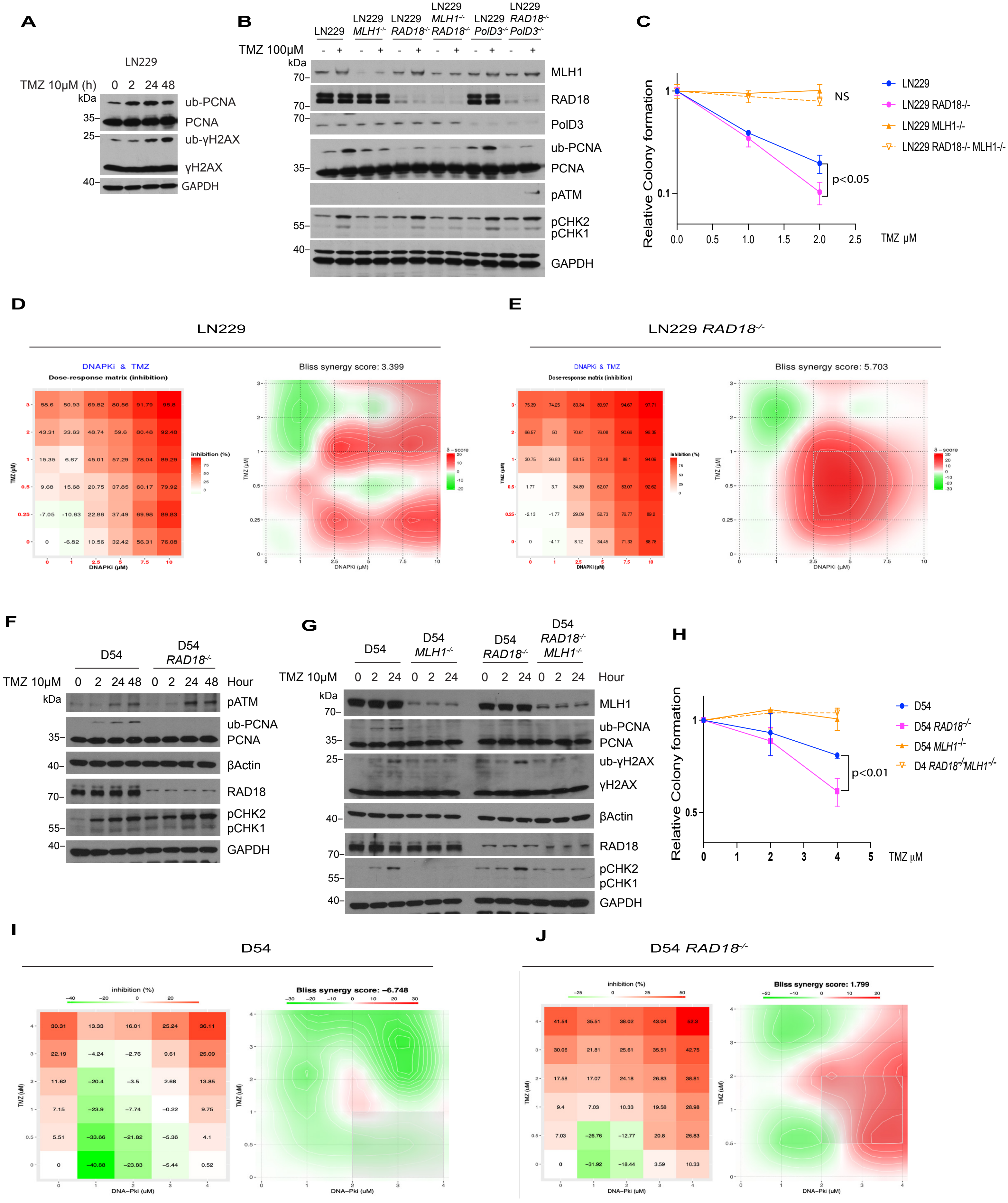

Supplementary Figure 7. RAD18 promotes TMZ-tolerance in GBM cell lines.

(A)-(E)* GBM cell line LN229: Immunoblot showing TMZ induced PCNA mono-ubiquitination and DDR markers in LN229 with time course (A) and *MLH1*$^{-/-}$, *RAD18*$^{-/-}$, *MLH1*$^{-/-}$*&RAD18*$^{-/-}$, *PolD3*$^{-/-}$ and *PolD3*$^{-/-}$*&RAD18*$^{-/-}$ (B). (C) Clonogenic survival assays showing TMZ-sensitivities of WT, *RAD18*$^{-/-}$, *MLH1*$^{-/-}$, and *RAD18*$^{-/-}$*MLH1*$^{-/-}$ LN229 cells. Cultures received a single treatment with TMZ daily for 5 days. Dose response matrices and synergy heatmaps showing effects of pairwise combinations of TMZ with DNA-PKi on inhibition of viability in *RAD18*$^{+/+}$ (D) and *RAD18*$^{-/-}$ (E) LN229 cells. **(F)-(J)** GBM cell line D54: Immunoblot showing TMZ induced PCNA mono-ubiquitination and DDR markers in D54 WT and *RAD18*$^{-/-}$ with time course (F) and *MLH1*$^{-/-}$, *RAD18*$^{-/-}$, *MLH1*$^{-/-}$*&RAD18*$^{-/-}$ (G). (H) Clonogenic survival assays showing TMZ-sensitivities of WT, *RAD18*$^{-/-}$, *MLH1*$^{-/-}$, and *RAD18*$^{-/-}$*MLH1*$^{-/-}$ D54 cells. Cultures received a single treatment with TMZ daily for 5 days. Dose response matrices and synergy heatmaps showing effects of pairwise combinations of TMZ with DNA-PKi on inhibition of viability in *RAD18*$^{+/+}$ (I) and *RAD18*$^{-/-}$ (J) D54 cells. All data points represent mean ± SD; p values were determined by two-sided t test (C,H).

*Note: The 10 and 100 μM doses of TMZ used for experiments shown in panels (A) and (B) exceed the lethal concentrations shown in the clonogenicity assay in panel (C). Relatively high TMZ concentrations were used in (A) and (B), because the induction of pCHK1 and mono-ubiquitinated PCNA at TMZ doses corresponding to the IC20 was undetectable due to the sensitivity limits of these immunoblotting experiments. In panels (A) and (G), and in several other supplementary figures in this report, a ubiquitinated species of γH2AX is indicated on immunoblots. Changes in levels of mono-ubiquitinated γH2AX typically track closely with fold changes in non-ubiquitinated γH2AX and provide a good surrogate marker for DSB. However, mono-ubiquitinated γH2AX levels are typically far less abundant than those of non-ubiquitinated γH2AX and do not generate saturated signals in immunoblotting experiments. Thus differences in levels of mono-ubiquitinated γH2AX between experimental samples can be quantified and interpreted more reliably than differences in γH2AX.

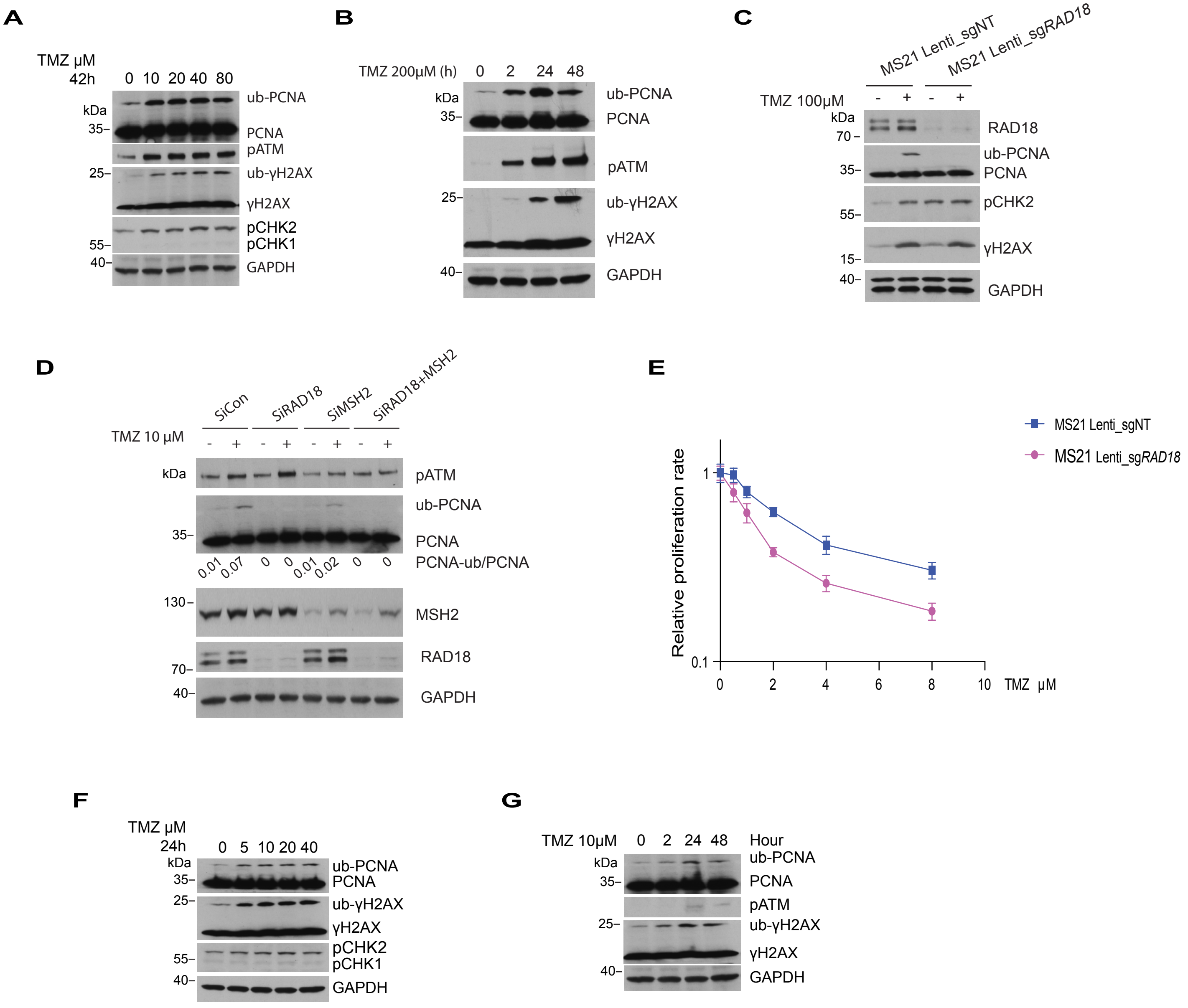

Supplementary Figure 8. RAD18 promotes TMZ-tolerance in GBM patient-derived line.

(A)-(E) low-passage patient-derived line MS21: Immunoblot showing TMZ induced PCNA mono-ubiquitination and DDR markers in MS21 with increasing dose **(A)** and time course **(B)**. Immunoblot showing both RAD18 and MSH2 mediate TMZ induced PCNA mono-ubiquitination. Lenti-CRISPR-Cas9 **(C)** and small interfering RNA **(D)** were used to deplete RAD18 and MSH2 genes. TMZ induced PCNA mono-ubiquitination. **(E)** Cell proliferation assays showing TMZ-sensitivities of MS21 Lenti-CRISPR-Cas9 RAD18 knockout and control pool cells. Cultures received a single treatment with TMZ daily for 5 days. All data points represent the mean ± SD of five replications. **(F&G)** high-passage patient-derived GBM stem cell line GBM8: Immunoblot showing TMZ induced PCNA mono-ubiquitination and DDR markers in GBM8 with increasing dose **(F)** and time course **(G)**.

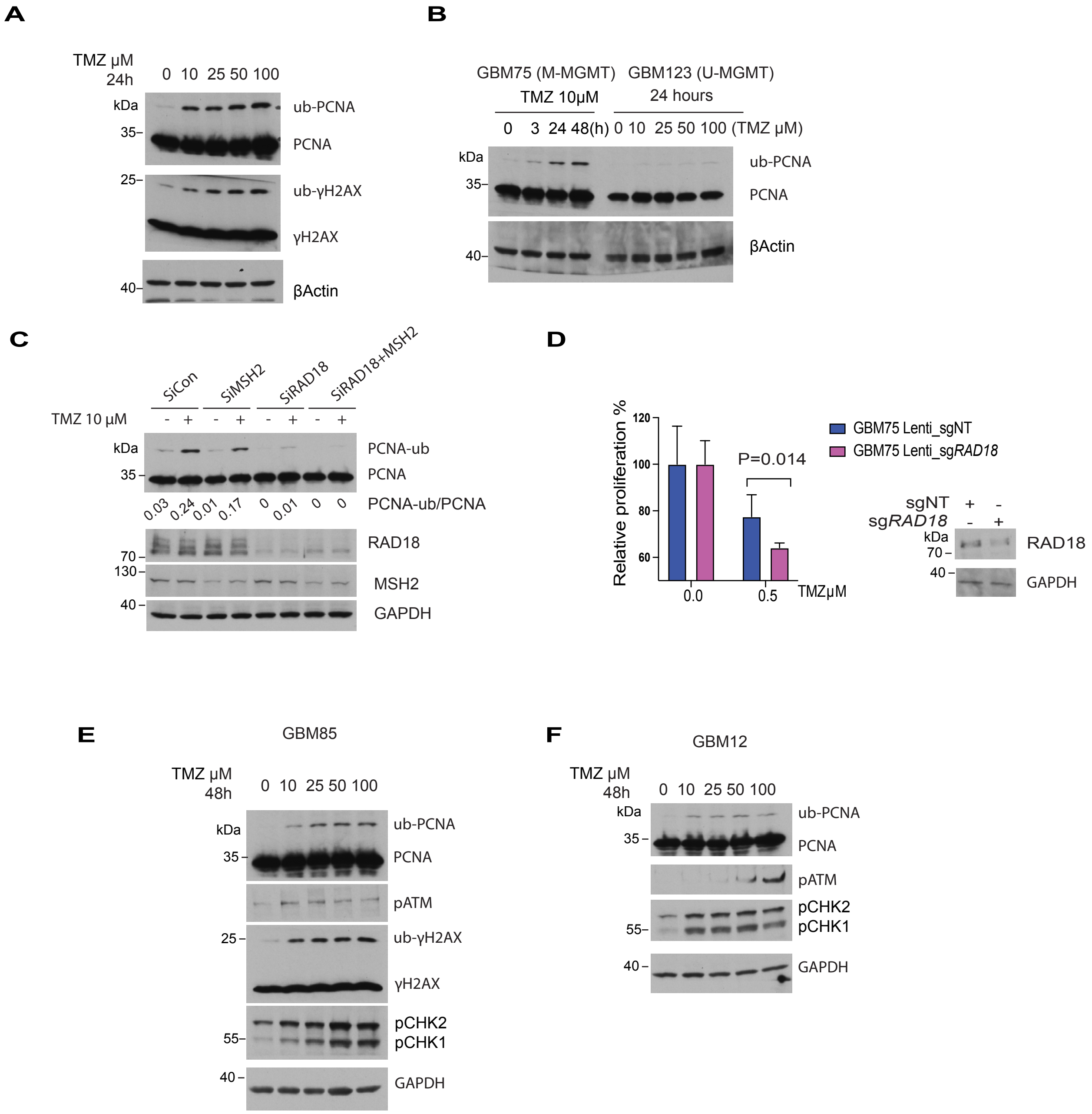

Supplementary Figure 9. RAD18 promotes TMZ-tolerance in GBM patient-derived xenografts (PDXs).

(A-D) Mayo clinic PDX adherent cell lines: GBM75 (methylation of MGMT gene promoter) and GBM123 (unmethylation of MGMT gene promoter). Immunoblot showing TMZ induced PCNA mono-ubiquitination and DDR markers in GBM75 with increasing dose **(A)**, time course (**B** left) and no TLS activation in GBM123 with increasing dose (**B** right). **(C)** Immunoblot showing both RAD18 and MSH2 mediate TMZ induced PCNA mono-ubiquitination. Small interfering RNA were used to deplete RAD18 and MSH2 genes. **(D)** Cell proliferation assays showing TMZ-sensitivities of GBM75 Lenti-CRISPR-Cas9 RAD18 knockout and control pool cells. Cultures received a single treatment with TMZ daily for 5 days. Expression level of RAD18 in knockout and control cells indicates the partial knockout. All data points represent the mean ± SD of five replications, p values were determined by two-sided t test. **(E&F)** Mayo clinic PDX stem cell lines. Immunoblot showing TMZ induced PCNA mono-ubiquitination and DDR markers in GBM85 **(E)** and GBM12 **(F)** with increasing dose.

1. **Design targeting sites**

Target sequence(20nt) PAM

NNNNNNNNNNNNNNNNNNNNNGG

2. **Oligos to synthesis**

5′ Franking sequence		3′ Franking sequence
GTGGAAAGGACGAAACACCG	NNNNNNNNNNNNNNNNNNNN	GTTTTAGAGCTAGAAATAGCAAGTTAAAATAAGG

3. **Clone oligos to LentiCRISPRv2-DD-Cas9-BSD vector by using 017_ArrayF and 018_ArrayR primers**

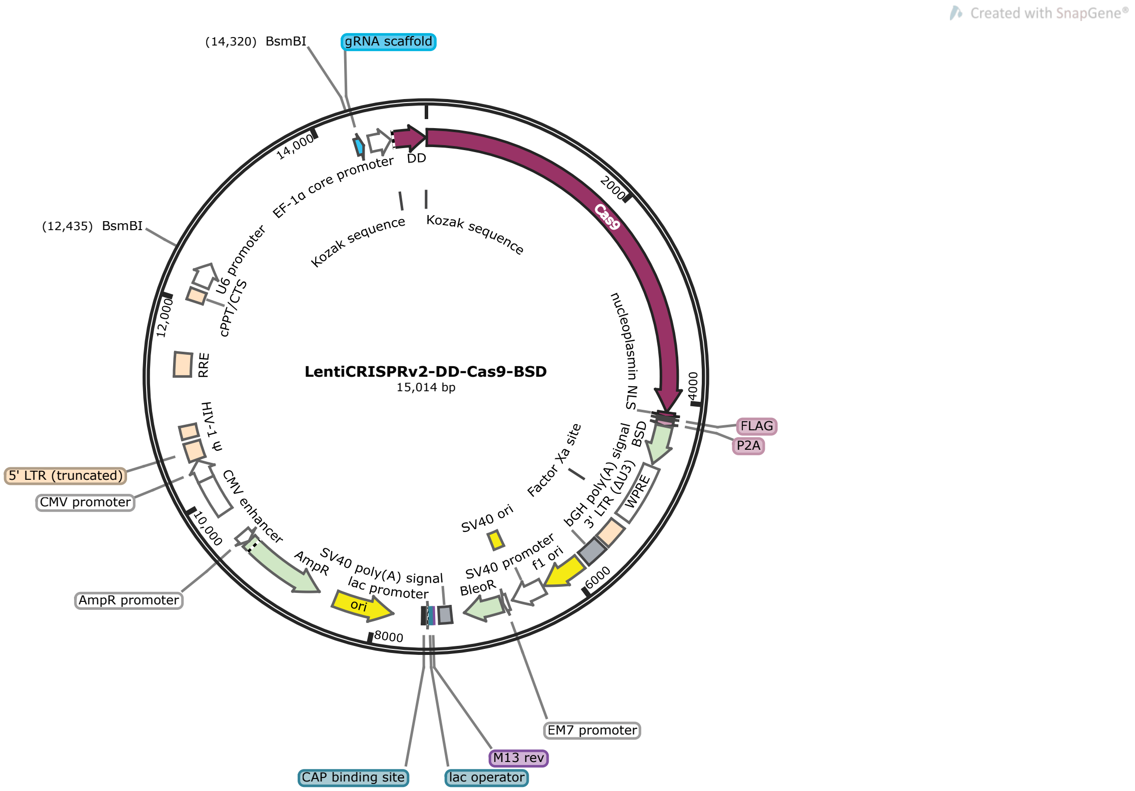

4. **Lentivirus packing**

Supplementary Figure 10. Summary of sgRNA library design and lentiviral vector used to screen for DDR-dependencies of TMZ-treated GBM cells.

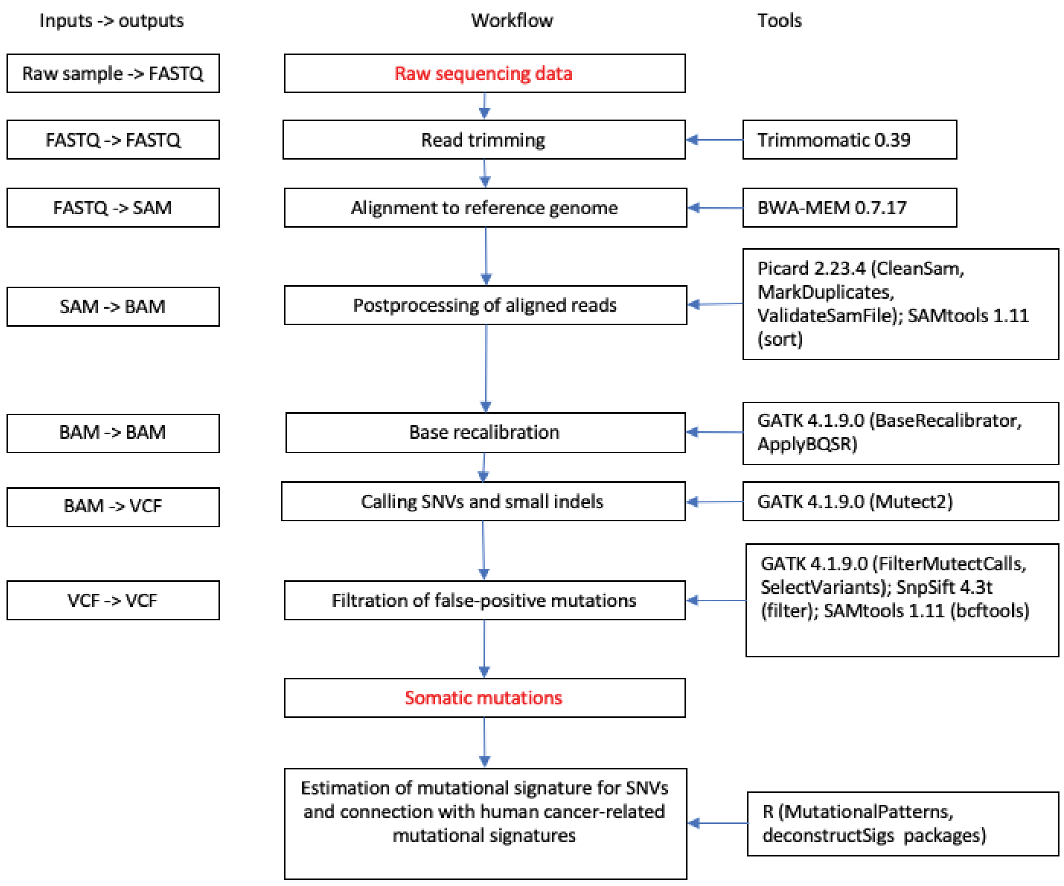

Supplementary Figure 11. Workflow of whole-exome sequencing data analysis.

Supplementary References

1 Tang, Z., Kang, B., Li, C., Chen, T. & Zhang, Z. GEPIA2: an enhanced web server for large-scale expression profiling and interactive analysis. *Nucleic Acids Res* **47**, W556-W560, doi:10.1093/nar/gkz430 (2019).
