# Supplementary material for: Trans-lesion synthesis and mismatch repair pathway crosstalk defines chemoresistance and hypermutation mechanisms in glioblastoma
Source: Nat Commun. 2024 Mar 4;15:1957. doi: 10.1038/s41467-024-45979-5 (PMC10912752; doi:10.1038/s41467-024-45979-5)
Supplement: Supplementary file 3 — Description of Additional Supplementary Files [file 41467_2024_45979_MOESM3_ESM.pdf]

Description of Additional Supplementary Files

File Name: Supplementary Data 1
Description: DDRlib_guide RNA

File Name: Supplementary Data 2
Description: DDR Screen sgRNA counts

File Name: Supplementary Data 3
Description: DDR Screen Gene list

File Name: Supplementary Data 4
Description: DNA Repair pathways core Gene List

File Name: Supplementary Data 5
Description: Pathway heatmap data

File Name: Supplementary Data 6
Description: WES SNV counts

File Name: Supplementary Data 7
Description: COSMIC_signaturev2_counts

File Name: Supplementary Data 8
Description: Patient clinical data

File Name: Supplementary Data 9
Description: Oligo sequence

File Name: Supplementary Data 10
Description: Cell lines STR profilling report

File Name: Supplementary Data 11
Description: Antibody information
