# Supplementary material for: Trans-lesion synthesis and mismatch repair pathway crosstalk defines chemoresistance and hypermutation mechanisms in glioblastoma
Source: Nat Commun. 2024 Mar 4;15:1957. doi: 10.1038/s41467-024-45979-5 (PMC10912752; doi:10.1038/s41467-024-45979-5)
Supplement: Supplementary file 14 — Supplementary Data 11 [file 41467_2024_45979_MOESM14_ESM.docx]

| Antibodies (Clone numbers) | Dilutions | Company Names | Catalog Numbers |
| --- | --- | --- | --- |
| Rabbit polyclonal anti- β-Actin | 1:5000 | Santa Cruz Biotechnology | Cat# sc-130656, RRID:AB_2223228 |
| Rabbit monoclonal anti- phospho-Chk1 (Ser345) | 1:1000 | Cell Signaling Technology | Cat# 2348, RRID:AB_331212 |
| Rabbit polyclonal anti- phospho-Chk2 (Thr68) | 1:1000 | Cell Signaling Technology | Cat# 2661, RRID:AB_331479 |
| Rabbit polyclonal anti- phospho-cdc2(Tyr15) | 1:1000 | Cell Signaling Technology | Cat# 9111, RRID:AB_331460 |
| Mouse monoclonal anti-phospho-H2A.X(Ser139) (JBW301) | 1:5000 | Millipore | Cat# 05-636, RRID:AB_309864 |
| Rabbit polyclonal anti- phospho-H3(Ser10) | 1:200 | Millipore | Cat# 06-570, RRID:AB_2315135 |
| Mouse monoclonal anti- Mitosin | 1:100 | BD Biosciences | Cat# 610768, RRID:AB_398091 |
| Mouse monoclonal anti- GAPDH (6C5) | 1:5000 | Santa Cruz Biotechnology | Cat# sc-32233, RRID:AB_627679 |
| Mouse monoclonal anti- RPA34 | 1:1000 | Millipore | Cat# NA19L, RRID:AB_565123 |
| Mouse monoclonal anti- PCNA (PC10) | 1:500 | Santa Cruz Biotechnology | Cat# sc-56, RRID:AB_628110 |
| Rabbit polyclonal anti- RAD18 | 1:500-3000 | Bethyl | Cat# A301-340A, RRID:AB_937974 |
| Rabbit polyclonal anti- Polη | 1:1000 | Bethyl | Cat# A301-231A, RRID:AB_890600 |
| Rabbit polyclonal anti- Polκ | 1:1000 | Bethyl | Cat# A301-977A, RRID:AB_1548020 |
| Rabbit polyclonal anti- Polι | 1:1000 | Bethyl | Cat# A301-304A, RRID:AB_937818 |
| Rabbit polyclonal anti-phaspho-RPA32(Ser33) | 1:1000 | Bethyl | Cat# A300-246A, RRID:AB_2180847 |
| Rabbit polyclonal anti-MGMT | 1:1000 | GeneTex | Cat# GTX110551, RRID:AB_1950916 |
| Mouse monoclonal anti-phospho-ATM(Ser1981) (10H11.E12) | 1:500 | Santa Cruz Biotechnology | Cat# sc-47739, RRID:AB_781524 |
| Mouse monoclonal anti- MSH2 (GB12) | 1:1000 | Millipore | Cat# NA26 RRID:AB_2144811 |
| Rabbit monoclonal anti-MLH1 | 1:1000 | Abcam | Cat# ab92312 RRID:AB_2049968 |
| Rabbit polyclonal anti- GFP | 1:1000 | Molecular Probes | Cat# A-11122, RRID:AB_221569 |
| Goat anti-Rabbit IgG Heavy and Light Chain Antibody HRP Conjugated | 1:5000 | Bethyl | Cat# A120-101P, RRID:AB_67264 |
| Goat anti-Mouse IgG Heavy and Light Chain Antibody HRP Conjugated | 1:5000 | Bethyl | Cat# A90-116P, RRID:AB_ 67183 |
| Alexa Fluor 555 donkey anti-Mouse IgG | 1:400 | Thermo Fisher Scientific | Cat# A-31570, RRID:AB_2536180 |
| Alexa Fluor 488 donkey anti-Rabbit IgG | 1:400 | Thermo Fisher Scientific | Cat#A21206, RRID:AB_2535792 |
| Alexa Fluor 647 donkey anti-Mouse IgG | 1:400 | Thermo Fisher Scientific | Cat# A-31571, RRID:AB_162542 |
