# Supplementary material for: Trans-lesion synthesis and mismatch repair pathway crosstalk defines chemoresistance and hypermutation mechanisms in glioblastoma
Source: Nat Commun. 2024 Mar 4;15:1957. doi: 10.1038/s41467-024-45979-5 (PMC10912752; doi:10.1038/s41467-024-45979-5)
Supplement: Supplementary file 15 — Reporting Summary [file 41467_2024_45979_MOESM15_ESM.pdf]

nature portfolio

Corresponding author(s): Yang Yang and Cyrus Vaziri

Last updated by author(s): Jan 27, 2024

Reporting Summary

Nature Portfolio wishes to improve the reproducibility of the work that we publish. This form provides structure for consistency and transparency in reporting. For further information on Nature Portfolio policies, see our Editorial Policies and the Editorial Policy Checklist.

Statistics

For all statistical analyses, confirm that the following items are present in the figure legend, table legend, main text, or Methods section.

n/a	Confirmed	
☐	☒	The exact sample size (*n*) for each experimental group/condition, given as a discrete number and unit of measurement
☒	☐	A statement on whether measurements were taken from distinct samples or whether the same sample was measured repeatedly
☐	☒	The statistical test(s) used AND whether they are one- or two-sided<br>*Only common tests should be described solely by name; describe more complex techniques in the Methods section.*
☒	☐	A description of all covariates tested
☐	☒	A description of any assumptions or corrections, such as tests of normality and adjustment for multiple comparisons
☐	☒	A full description of the statistical parameters including central tendency (e.g. means) or other basic estimates (e.g. regression coefficient) AND variation (e.g. standard deviation) or associated estimates of uncertainty (e.g. confidence intervals)
☐	☒	For null hypothesis testing, the test statistic (e.g. *F*, *t*, *r*) with confidence intervals, effect sizes, degrees of freedom and *P* value noted<br>*Give P values as exact values whenever suitable.*
☒	☐	For Bayesian analysis, information on the choice of priors and Markov chain Monte Carlo settings
☒	☐	For hierarchical and complex designs, identification of the appropriate level for tests and full reporting of outcomes
☒	☐	Estimates of effect sizes (e.g. Cohen's *d*, Pearson's *r*), indicating how they were calculated

Our web collection on statistics for biologists contains articles on many of the points above.

Software and code

Policy information about availability of computer code

Data collection	ZEN image software and Fusion 2.4 (ANDOR) were used to collect images.
Data analysis	DDR screen data analysis code is available by https://github.com/CHENGHAO-WANG/radarChart/tree/main; Mutagenesis assay code is available by https://github.com/jitonglou/RAD18_TMZ_data. BWA package (version 0.7.17), Picard 2.23.4 (http://broadinstitute.github.io/picard), SAMtools 1.11,bcftools in SAMtools, IndexFeatureFile, BaseRecalibrator, ApplyBQSR and Mutect2, FilterMutectCalls tool in GATK 4.1.9.0, SnpSift 4.3t, Graphpad Prism 10, R Studio, ImageJ FIJI, ColonyArea package, C6 Plus and Imaris10 had been used for data analysis.

For manuscripts utilizing custom algorithms or software that are central to the research but not yet described in published literature, software must be made available to editors and reviewers. We strongly encourage code deposition in a community repository (e.g. GitHub). See the Nature Portfolio guidelines for submitting code & software for further information.

Data

Policy information about availability of data

All manuscripts must include a data availability statement. This statement should provide the following information, where applicable:

- Accession codes, unique identifiers, or web links for publicly available datasets
- A description of any restrictions on data availability
- For clinical datasets or third party data, please ensure that the statement adheres to our policy

The whole exom sequencing (WES) data used in this study are available in the NCBI Sequence Read Archive (SRA) under accession code PRJNA901961 (http://www.ncbi.nlm.nih.gov/bioproject/901961). The homo sapiens reference genome GRCh37/hg19 had used for sequence mapping. The protein coding exon region

database (https://earray.chem.agilent.com/suredesign/) and Agilent SureSelect DNA - SureSelect Human All Exon V6 had been sued to map the mutation calls. Known simple nucleotide polymorphisms (dbSNP Build 151) reported in the GRCh37/hg19 background by NCBI (https://ftp.ncbi.nih.gov/snp/organisms/human_9606_b151_GRCh37p13/VCF/common_all_20180423.vcf.gz) were used to remove the preexisting mutations from analysis.

Research involving human participants, their data, or biological material

Policy information about studies with human participants or human data. See also policy information about sex, gender (identity/presentation), and sexual orientation and race, ethnicity and racism.

Reporting on sex and gender	*Use the terms sex (biological attribute) and gender (shaped by social and cultural circumstances) carefully in order to avoid confusing both terms. Indicate if findings apply to only one sex or gender; describe whether sex and gender were considered in study design; whether sex and/or gender was determined based on self-reporting or assigned and methods used.* *Provide in the source data disaggregated sex and gender data, where this information has been collected, and if consent has been obtained for sharing of individual-level data; provide overall numbers in this Reporting Summary. Please state if this information has not been collected.* *Report sex- and gender-based analyses where performed, justify reasons for lack of sex- and gender-based analysis.*
Reporting on race, ethnicity, or other socially relevant groupings	*Please specify the socially constructed or socially relevant categorization variable(s) used in your manuscript and explain why they were used. Please note that such variables should not be used as proxies for other socially constructed/relevant variables (for example, race or ethnicity should not be used as a proxy for socioeconomic status).* *Provide clear definitions of the relevant terms used, how they were provided (by the participants/respondents, the researchers, or third parties), and the method(s) used to classify people into the different categories (e.g. self-report, census or administrative data, social media data, etc.)* *Please provide details about how you controlled for confounding variables in your analyses.*
Population characteristics	*Describe the covariate-relevant population characteristics of the human research participants (e.g. age, genotypic information, past and current diagnosis and treatment categories). If you filled out the behavioural & social sciences study design questions and have nothing to add here, write "See above."*
Recruitment	*Describe how participants were recruited. Outline any potential self-selection bias or other biases that may be present and how these are likely to impact results.*
Ethics oversight	*Identify the organization(s) that approved the study protocol.*

Note that full information on the approval of the study protocol must also be provided in the manuscript.

Field-specific reporting

Please select the one below that is the best fit for your research. If you are not sure, read the appropriate sections before making your selection.

☒ Life sciences ☐ Behavioural & social sciences ☐ Ecological, evolutionary & environmental sciences

For a reference copy of the document with all sections, see nature.com/documents/nr-reporting-summary-flat.pdf

Life sciences study design

All studies must disclose on these points even when the disclosure is negative.

Sample size	No sample size calculation was performed. At least 3 technical and/or biological replicates for each sample was done. Multi-institute cohort of TMZ-treated 73 recurrent GBM (rGBM) patients mutation and RNAseq data were used in this study. We included all the available samples into analysis.
Data exclusions	In the GBM patient cohort analysis, data from POLE-mutated samples were excluded from analysis based on previous work implicating POLE alterations in GBM hypermutation.
Replication	Screen had performed once with 3 biological replicates per condition; Mutagenesis had perform once with 6 independent single clone samples per condition were picked up for WES analysis; Colony formation experiments had performed at least twice with triplicates per condition; Experiments to build dose response matrices and synergy heatmap had performed once with triplicates per condition; Experiments for immunoblot performed at least twice independently with similar results. Patient samples were limited, therefore no repeat for the patient data analysis.
Randomization	No. We included all the available samples.
Blinding	No. The clinical information are available.

Reporting for specific materials, systems and methods

We require information from authors about some types of materials, experimental systems and methods used in many studies. Here, indicate whether each material, system or method listed is relevant to your study. If you are not sure if a list item applies to your research, read the appropriate section before selecting a response.

Materials & experimental systems

n/a	Involved in the study	
☐	☒	Antibodies
☐	☒	Eukaryotic cell lines
☒	☐	Palaeontology and archaeology
☐	☒	Animals and other organisms
☐	☒	Clinical data
☒	☐	Dual use research of concern
☐	☐	Plants

Methods

n/a	Involved in the study	
☒	☐	ChIP-seq
☐	☒	Flow cytometry
☒	☐	MRI-based neuroimaging

Antibodies

Antibodies used

Rabbit polyclonal anti- beta-Actin 1:5000 Santa Cruz Biotechnology Cat# sc-130656, RRID:AB_2223228
Rabbit monoclonal anti- phospho-Chk1 (Ser345) 1:1000 Cell Signaling Technology Cat# 2348, RRID:AB_331212
Rabbit polyclonal anti- phospho-Chk2 (Thr68) 1:1000 Cell Signaling Technology Cat# 2661, RRID:AB_331479
Rabbit polyclonal anti- phospho-cdc2(Tyr15) 1:1000 Cell Signaling Technology Cat# 9111, RRID:AB_331460
Mouse monoclonal anti-phospho-H2A.X(Ser139) 1:5000 Millipore Cat# 05-636, RRID:AB_309864
Rabbit polyclonal anti- phospho-H3(Ser10) 1:400 Millipore Cat# 06-570, RRID:AB_2315135
Mouse monoclonal anti- Mitosin BD Biosciences Cat# 610768, RRID:AB_398091
Mouse monoclonal anti- GAPDH 1:5000 Santa Cruz Biotechnology Cat# sc-32233, RRID:AB_627679
Mouse monoclonal anti- RPA34 1:1000 Millipore Cat# NA19L, RRID:AB_565123
Mouse monoclonal anti- PCNA 1:500 Santa Cruz Biotechnology Cat# sc-56, RRID:AB_628110
Rabbit polyclonal anti- RAD18 1:100 Bethyl Cat# A301-340A, RRID:AB_937974
Rabbit polyclonal anti- Polh 1:1000 Bethyl Cat# A301-231A, RRID:AB_890600
Rabbit polyclonal anti- Polk 1:1000 Bethyl Cat# A301-977A, RRID:AB_1548020
Rabbit polyclonal anti- Poli 1:1000 Bethyl Cat# A301-304A, RRID:AB_937818
Rabbit polyclonal anti-phaspho-RPA32(Ser33) 1:1000 Bethyl A300-246A, RRID:AB_2180847
Rabbit polyclonal anti-MGMT 1:1000 GeneTex Cat# GTX110551, RRID:AB_1950916
Mouse monoclonal anti-phospho-ATM(Ser1981) 1:500 Santa Cruz Biotechnology Cat# sc-47739, RRID:AB_781524
Mouse monoclonal anti- MSH2 1:1000 Millipore Cat# NA26 RRID:AB_2144811
Rabbit monoclonal anti-MLH1 1:1000 Abcam Cat# ab92312 RRID:AB_2049968
Rabbit polyclonal anti- GFP 1:1000 Molecular Probes Cat# A-11122, RRID:AB_221569
Goat anti-Rabbit IgG Heavy and Light Chain Antibody HRP Conjugated 1:5000 Bethyl Cat# A120-101P, RRID:AB_67264
Goat anti-Mouse IgG Heavy and Light Chain Antibody HRP Conjugated 1:5000 Bethyl Cat# A90-116P, RRID:AB_ 67183
Alexa Fluor 555 donkey anti-Mouse IgG 1:400 Thermo Fisher Scientific Cat# A-31570, RRID:AB_2536180
Alexa Fluor 488 donkey anti-Rabbit IgG 1:400 Thermo Fisher Scientific Cat#A21206, RRID:AB_2535792
Alexa Fluor 647 donkey anti-Mouse IgG 1:400 Thermo Fisher Scientific Cat# A-31571, RRID:AB_162542

Validation

Our lab has been study DNA repair and TLS pathway for more than a decade. The routine DNA repair, cell cycle and TLS protein such as PCNA, RAD18, Polh, Polk, Poli, RPA32, pRPA32, gH2AX, pChK1, pChK2, pCdc2, pATM, MSH2, MLH1, pH3 and CyclinE antibodies had been used in many of our publications with appropriate positive and negative controls such as knockdown and knockout cell lines, with/without DNA damage agents, or with/without cell cycle arresting. Mitosin and MGMT are first used antibodies in this study. We had validated Mitosin by FACS and Immunofluorescence imaging and MGMT by LN18 (MGMT+) and U373 (MGMT-) cell line western blotting. The internal control antibodies such as beta-Actin and GAPDH and the Alexa Fluor antibodies are wildly used and highly cited in numerous publication.

Eukaryotic cell lines

Policy information about cell lines and Sex and Gender in Research

Cell line source(s)

U373, U87, D54, LN229 and LN18 were purchased from the American Type Culture Collection (ATCC). NHA and NHA-RAS cell lines were gifted by Dr. Russell O. Pieper, University of California San Francisco, GBM8 were gifted by Dr. Hiroaki Wakimoto from Massachusetts General Hospital, MS21 were generated by Hingtgen lab, GBM PDXs (GBM12, GBM75 GBM85 and GBM123) were shared by Dr. Jann Sarkaria from Mayo Clinic

Authentication

We perform short tandem repeat (STR) profiling on U373, U87 and LN18 to confirm the ATCC GBM lines. We didn't performed any authentication for the gift cell lines. Loss of RAD18, MLH1, MSH2, POLK and POLD3 function cell lines were determined as described in the methods by TIDE assay and western blot.

Mycoplasma contamination

All cells are routinely tested for and found to be mycoplasma free as described in the Methods

Commonly misidentified lines
(See ICLAC register)

The ATCC stock for U-373 MG was in turn found to be identical to U-251 MG

Animals and other research organisms

Policy information about studies involving animals; ARRIVE guidelines recommended for reporting animal research, and Sex and Gender in Research

Laboratory animals	Sprague-Dawley rat, age: P8
Wild animals	No wild animals were used in the study.
Reporting on sex	We worked to ensure sex balance in the selection of non-human subjects.
Field-collected samples	No field collected samples were used in the study.
Ethics oversight	All work was approved by the Institutional Animal Care and Use Committee at the University of North Carolina-Chapel Hill

Note that full information on the approval of the study protocol must also be provided in the manuscript.

Clinical data

Policy information about clinical studies

All manuscripts should comply with the ICMJEguidelines for publication of clinical research and a completedCONSORT checklist must be included with all submissions.

Clinical trial registration	*Provide the trial registration number from ClinicalTrials.gov or an equivalent agency.*
Study protocol	*Note where the full trial protocol can be accessed OR if not available, explain why.*
Data collection	We reanalyzed the patient WES and RNAseq data from Wang et al Nat. Gen. 2016 paper
Outcomes	*Describe how you pre-defined primary and secondary outcome measures and how you assessed these measures.*

Plants

Seed stocks	*Report on the source of all seed stocks or other plant material used. If applicable, state the seed stock centre and catalogue number. If plant specimens were collected from the field, describe the collection location, date and sampling procedures.*
Novel plant genotypes	*Describe the methods by which all novel plant genotypes were produced. This includes those generated by transgenic approaches, gene editing, chemical/radiation-based mutagenesis and hybridization. For transgenic lines, describe the transformation method, the number of independent lines analyzed and the generation upon which experiments were performed. For gene-edited lines, describe the editor used, the endogenous sequence targeted for editing, the targeting guide RNA sequence (if applicable) and how the editor was applied.*
Authentication	*Describe any authentication procedures for each seed stock used or novel genotype generated. Describe any experiments used to assess the effect of a mutation and, where applicable, how potential secondary effects (e.g. second site T-DNA insertions, mosiacism, off-target gene editing) were examined.*

Flow Cytometry

Plots

Confirm that:

- [x] The axis labels state the marker and fluorochrome used (e.g. CD4-FITC).
- [x] The axis scales are clearly visible. Include numbers along axes only for bottom left plot of group (a 'group' is an analysis of identical markers).
- [x] All plots are contour plots with outliers or pseudocolor plots.
- [x] A numerical value for number of cells or percentage (with statistics) is provided.

Methodology

Sample preparation	Cells were trypsinized and fixed in paraformaldehyde or ethanol as indicated in the text
Instrument	ACCURI C6 plus
Software	C6 Plus Analysis Software
Cell population abundance	Cells were analyzed by Western Blot as described in the text.
Gating strategy	FL2H/FL2A gates define single cell population. For each condition. FL1A channel for FICT, Alex Flour 488, GPF, FL2A channel

for Alex Flour 555, mCherry, RFP and PI, FL4A for Alex Flour 647

☒ Tick this box to confirm that a figure exemplifying the gating strategy is provided in the Supplementary Information.
